# Efficacy of Music Therapy in Treatment for the Patients with Alzheimer's Disease

**DOI:** 10.1155/2012/531646

**Published:** 2012-09-26

**Authors:** H. Fukui, A. Arai, K. Toyoshima

**Affiliations:** ^1^Department of Education, Nara University of Education, Takabatake, Nara 630-8528, Japan; ^2^Ongakunomori Nonprofit Organization, 1-12 Kobocho, Saidaiji, Nara 631-0827, Japan

## Abstract

We report that music therapy is effective in the treatment of Alzheimer's disease. We found that the secretion of 17*β*-estradiol and testosterone, hormones that are supposed to have preventive effects on Alzheimer's disease, is significantly increased by music therapy. During the sessions, patients with Alzheimer's disease were allowed to listen to music and songs with verbal contact from the therapist. It was found that problematic behaviors such as poriomania (fugue) had decreased. Music therapy has the potential as an alternative treatment for adverse hormone replacement therapy.

## 1. Introduction

In both sexes, sex hormone levels decrease with aging. These reductions have been correlated to various symptoms in the elderly including diminished cognitive function, disturbance of memory, mind and mood, depression, and climacteric disturbance [1]. 

In an attempt to mitigate these symptoms, hormone replacement therapies are administered, for example, estrogen in females and androgen in males [2–4] and females [5, 6]. Even within the elderly population, sex hormone levels are lower in Alzheimer patients than in healthy counterparts [4, 7].

In Alzheimer's disease, the aging-related reductions in sex hormones, especially estrogen, represent a critical risk factor [8–10]. This is because estrogen protects the nerves and acts to control cell proliferation. Moreover, estrogen decreases the *β* amyloid peptide content in the neurons which is a typical pathologic finding in Alzheimer's disease [11, 12] and protects the body from neurotoxicity from *β* peptide [13, 14]. Estrogen has also shown to suppress the increase and deposition of *β*-amyloid and to prevent nerve cell damage [12, 15, 16]. In addition to these effects on amyloid metabolism, estrogen improves cognitive function and delays the onset of dementia by increasing cholinergic activity in the brain, stimulating axonal budding and dendrite formation and retarding cerebral arteriosclerosis [9]. Hence, estrogen replacement therapy has been recommended as a prophylaxis of Alzheimer's disease in particular for the elderly female patients with decreased estrogen levels [8, 17].

In actual clinical settings, estrogen is used to treat postmenopausal women with Alzheimer's disease and has shown its effect to improve their verbal memory and attention remarkably [18–20]. It has also shown that the onset of dementia is delayed significantly in elderly women who had been receiving estrogen for long periods than those who had not [21–23]. Another report shows that the incidence of Alzheimer's disease was significantly lower in estrogen recipients than in controls [24]. Estrogen has proved to be effective in the treatment and prevention for Alzheimer's disease [7].

In recent years, the nerve-protecting action of androgens, especially of testosterone, has been noticed attentively. A study of cortical neurons of cultured rats has shown that testosterone increases NGF (nerve growth factor) and p-75 nerve growth factor receptor and decreases *β* amyloid peptide in mouse model of Alzheimer's disease [25, 26]. Similarly, in the human neurons, it has been reported that androgens, such as testosterone, enanthate, methyl testosterone and epitestosterone, suppress nerve apoptosis and protect the nerves [8, 17]. Since the action of testosterone is especially distinct in the portions of the brain that control cognition and memory, the relationship between this hormone and cognitive function has also been investigated. According to Yaffe et al. [27], with the increase in testosterone levels, cognitive test score increases in elderly males. Therefore, prescription of testosterone supplements for males has been suggested as possibly reducing the risk of diminishment of cognitive ability, a prodrome of Alzheimer's disease. Hence, the reductions in testosterone levels with aging represent a risk factor of Alzheimer's disease [8, 9]. However, hormone replacement therapy has its drawbacks and is not used today. This topic is discussed later (Section 4).

Recent studies revealed that music is closely associated with hormones which govern the emotion and human behavior, especially with steroid hormones including sex hormones. It has been shown that there is a correlation between spatial ability or music ability and testosterones [28], and listening to music has effects on testosterones and cortisol [29–31]. The correlation between musical ability and spatial cognition has long been known [32–34]. Many studies have investigated the relationship of musical ability to spatial perception and cognition in human being. The assumption that some correlation exists between musical ability and steroid hormones seems to be appropriate. In fact, Hassler discovered that the relationship between T and musical ability (music composition) resembles the one between T and other forms of spatial perception and cognition [33, 35].

 Furthermore, the relationship between music and steroid hormones is not limited to musical ability. In the field of behavioral endocrinology and neuroendocrinology, many studies have documented that musical stimulation (listening) affects various biochemical substances [36–38]. In particular, many studies-based findings on C. Experiment had shown that listening to music is effective in alleviating and decreasing stress. In many studies, stress reduction due to music listening has been attributed to reductions in C [39, 40]. It also has been noted that listening to music alters levels of T (increase and decrease) [41, 42]. The research reported that musical activities (listening and playing) adjust steroid secretion in elderly individuals and are likely to alleviate psychological states such as anxiety and tension. Moreover, levels of steroids changing in both directions, increasing in subjects with low hormone levels, and decreasing in subjects with high hormone levels were found [43]. Additionally, there has been a report that listening to the music enhances cognitive recovery of mood after middle cerebral artery stroke, and listening to the music during the early poststroke stage can enhance cognitive recovery and prevent negative mood [44]. Also, music-supported therapy (MST) on patients who had an acute and chronic stroke could bring the neuroplastic changes in the neural circuit underlying audiomotor coupling [44].

## 2. Materials and Methods

In the present study, we monitored testosterone and 17 *β*-estradiol levels over time in patients with Alzheimer's disease stimulated with music, to determine whether music therapy has the potential as an alternative treatment for hormone replacement therapy, focusing on the fact that the hormones bearing a causative relation to the onset of Alzheimer's disease are also closely related to music.

Traditionally, studies of the efficacy of music therapy in patients with Alzheimer's disease have focused on changes in symptoms such as dementia most typically and other problematic behaviours: aggressive behaviors, depression, disturbance of mood, and decreased sociality [45–52]. In general, behavioral therapy has shown an alleviation on behavioral deterioration by enhancing the patient's social interactions, more specifically one to one interactions with carers, therapists, and others [49, 53]. In these studies, mitigation of symptoms was achieved but the mechanism of action remained unexplained.

In music therapy, whether the observed therapeutic effects are attributable to the music, the therapist, or their synergism is often obscure and unidentified. The study population comprised of six patients with an established diagnosis of Alzheimer's disease (6 females, ages ranging from 67 to 90 years, mean age 81.8 years) residing in a special nursing home for the elderly. Every subject's family or guardian had received the written informed consent before participating in this study based on the Declaration of Helsinki (1964). The patients were allocated with three conditions (within subjects designs)

1. The subjects had only been greeted and been questioned upon their health and mood by the therapist. There was no music involved in this condition.
2. 12 songs that had been selected in a preliminary survey were sung by the therapist. It was then used for the subjects to listen to.
3. Music therapy that comprises of (1) and  (2).

The session was carried out for the duration of a month, and each session took about an hour. Salivary hormone levels were measured before and after each session. The effects and differences on hormone levels were compared between the before and the after. The therapist contacted the subjects verbaly whose scenarios were formulated prior to the session. A total of 12 songs were selected on the basis of preference by each subject in a preliminary survey. Then, the therapist sang chosen songs without microphone accompanied by the keyboard sound from an amplified speaker. At the same time, each subject's behavior was evaluated with each condition for three consecutive days: a day before the session, a day of the session, and a day after the session.

Before starting the experiments, a survey was conducted on medical aspects: past history, medication status, and so forth, daily life: lifestyle (possible, but preferably life-style or life style or could use preference of life), daily activity dependence, extent of care, food preference, hobbies, personal relations, communication capability, personality, and other aspects of each subject. Additionally, they were asked of their experiences of performing the music, music-related activities in daily life. Regarding their hearing status, the subjects were examined to have acceptable auditory senses, provided that they retained hearing ability that permits them to have everyday life without difficulty even though the ability had been diminished naturally with aging. None of the subjects were on hormone replacement therapy and known of used any drug use that significantly influence steroid hormones. All subjects had already received music therapy for at least four consecutive months (4 years and 4 months at maximum, 4 months at minimum).

36 samples, 6 subjects × before the session and after session × three conditions were collected. Saliva samples were kept frozen at −20°C until assayed. Salivary 17 *β*-estradiol and testosterone levels were assayed in duplicate by EIA kit (Assay Designs, Inc.). The kit is used for the quantitative measurement of 17 *β*-estradiol (E) and testosterone (T). It occupies a monoclonal antibody to each hormone to bind in a competitive manner, and it targets in a sample or an alkaline phosphatase molecule which has hormones covalently attached to it. The established intra-assay coefficiency of E and T variance was 5.7% and 7.8%, respectively, and the interassay coefficient of variations for E and T is 6.2%, and 9.3%, respectively. The measured intraassay coefficient of variations for E and T was 5.3%, 6.2%, respectively, and the interassay coefficientof variations for E and T were 5.6%, and 7.4%, respectively.

## 3. Results

Regarding the influence of physical factors involved in the individual conditions, analysis of variance (ANOVA) and *t*-test were employed to determine whether there are differences in duration, tempo, and sound pressure of music between “listening to the music” and “music therapy.” As a result, no such differences were found (*F*(1,22) = 2.284, *P* = 0.1450; *F*(1,22) = 2.754, *P* = 0.1112; *t* = −1.475, *P* = 0.1462). Therefore, it can be concluded that there are no differences in physical factors of music conditions between “listening to the music listening” and “music therapy.”

**Figure 1:**
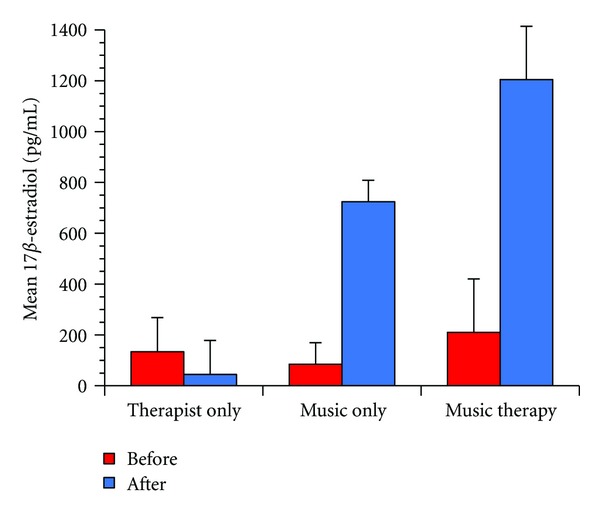
Changes in 17 *β*-estradiol concentrations. Changes in 17 *β*-estradiol concentrations in 6 patients with Alzheimer's disease at each experimental condition. Two-way ANOVA revealed that the main effect of group (*P* = 0.0389), main effect of changes in 17 *β*-estradiol level (*P* = 0.0026), and interaction with group (*P* = 0.0177) were significant.

The mean 17 *β*-estradiol level for the subjects was 253.539 pg/mL. Two-way ANOVA with more than one observation was conducted with “hormonal changes between before and after stimuli” and “group,” “listening to the music,” “therapist,” and “music therapy”—as variables. As a result, statistically significant differences were found in terms of “main effect of group” (*F*(2,9) = 4.760, *P* = 0.0389), “main effect of changes in 17 *β*-estradiol level” (*F*(1,9) = 16.987, *P* = 0.0026), and “interaction with group” (*F*(2,9) = 6.528, *P* = 0.0177). Although the 17 *β*-estradiol level increased after the “listening to the music,” the greatest increase was obtained after the “music therapy” (Figure 1). On the other hand, the 17 *β*-estradiol level decreased after the “therapist” conditioned intervention. A post hoc test (Fisher's PLSD) revealed a significantly increased 17 *β*-estradiol level for the “music therapy” condition as compared to the “therapist” condition (*P* = 0.0130).

**Figure 2:**
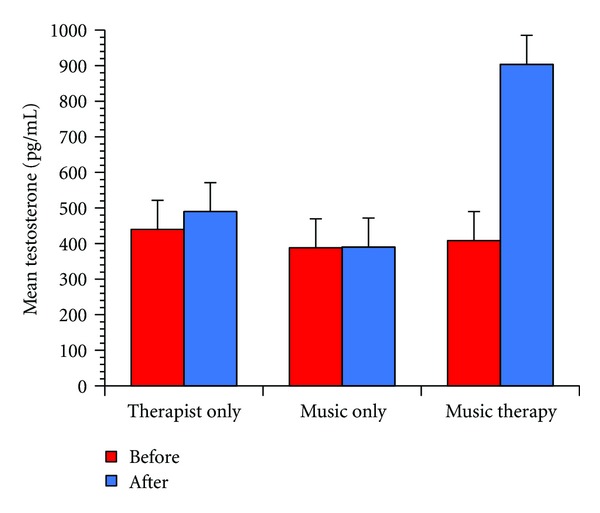
Changes in testosterone concentrations. Changes in testosterone concentrations in 6 patients with Alzheimer's disease at each experimental condition. Two-way ANOVA revealed that the main effect of group (*P* = 0.05), main effect of changes in testosterone level (*P* = 0.0066), and interaction with group (*P* = 0.0063) were significant.

The mean testosterone level for the subjects was 450.672 pg/mL. Two-way ANOVA with more than one observation was conducted with “group” under three conditions and “hormonal changes between before and after stimuli” as variables. As a result, statistically significant differences were found in terms of main effect of “group” (*F*(2,5) = 5.72, *P* = 0.05), main effect of testosterone level changes (*F*(1,5) = 19.9, *P* = 0.0066), and interaction with “group” (*F*(2,5) = 16.5, *P* = 0.0063). A post hoc test (Fisher's PLSD) revealed a significantly increased testosterone level for the “music therapy” condition as compared to the “music listening” condition (*P* = 0.0213) (Figure 2). Carers have reported that problematic behavior was decreased after the “music therapy” condition, and it lasted till a day after the session.

## 4. Discussion

These achieved results demonstrate that the “music therapy” conditionincreased the testosterone level significantly after stimuli in comparison to the other conditions. Also, the results suggest that problematic behavior can be reduced by music therapy.

Since behavioral therapy involves human relations, it is inevitable that the therapy is strongly dependent on social interactions [49, 51, 53]. In the present study, the effects of “music” and “therapist” were separately evaluated using endocrine indices. It showed that with patients with Alzheimer's disease at the initial stage, the greatest effect is obtained by “music therapy,” a combination of “therapist” and “listening to the music,” as opposed to the ones being employed alone. The hormones, 17*β*-estradiol and testosterone that served as indices in this study, have been reported to suppress the degeneration and diminishment of neurofibrils, which a typical character of Alzheimer's disease is supposed to be caused by. As it was seen in this study, the increases in 17*β*-estradiol and testosterone levels observed in the patients with Alzheimer's with diminishing hormones suggest that music therapy may contribute to decelerate the progression of Alzheimer's disease or even to delay its onset. It is considered that music therapy restores normal hormone levels and suppresses nerve cell damage and protects nerve cells, thus terminating the progression of Alzheimer's disease. 

In general, hormone replacement therapy (HRT) is expected to be highly effective in the prevention and the treatment for Alzheimer's disease. However, HRT is not applicable to all Alzheimer's patients as there is risk of causing adverse reactions such as invasive breast cancer, heart disease, and strokes [54]. In fact, there have been a report that adverse reactions were caused in Alzheimer's patients on HRT. The reactions include increased risks of carcinogenicity in females, feminization in males with estrogen replacement therapy, increased risks of prostatic cancer, elevated cholesterol levels, acne, alopecia, and other symptoms in males receiving testosterone replacement therapy [28, 55]. Therefore, there is a strong demand for a development of an appropriate treatment that can avoid causing such aversive and unwanted reactions [21, 56].

Based on this current study, music therapy seems to be an alternative that is more unlikely to cause risks of harmful reactions to the patients than HRT. The possibility of causing such risks is thought to be significantly lower when music therapy is used than when HRT is used. It is vital to note that no aversive reactions were induced by the music, except music epilepsy [57], which has been reported in a very small percentage of cases. For this reason, music therapy has a potential to become as a safe alternative treatment that is as effective as HRT but with lower prevalence of unwanted reactions. Moreover, music therapy can be expected to serve as an effective prophylaxis of Alzheimer's disease for the healthy elderly. Music therapy therefore has a potential to be an alternative to HRT hormone replacement therapy for the healthy elderly population.
